# PATHOPHYSIOLOGY, DIAGNOSIS AND TREATMENTOF DUMPING SYNDROME AND ITS RELATION TO BARIATRIC SURGERY

**DOI:** 10.1590/0102-6720201600S10028

**Published:** 2016

**Authors:** Yasmin da Silva CHAVES, Afrânio Côgo DESTEFANI

**Affiliations:** Faculdade de Ciências Biomédicas do Espírito Santo, Cariacica, ES, Brazil.

**Keywords:** Dumping syndrome, Digestive system diseases, Bariatric surgery

## Abstract

**Introduction:**

The dumping syndrome is frequent in bariatric surgery. It is probably the most common syndrome following partial or complete gastrectomy. Its prevalence in partial gastrectomy can reach up to 50%, thus it can be a significant complication arising from some types of bariatric surgeries.

**Objective:**

Critical analysis on dumping syndrome, its pathophysiology, diagnosis and treatment.

**Methods:**

A literature review was performed using the key words: 'dumping syndrome', 'bariatric surgery' and 'rapid dumping syndrome'. Inclusion criteria were: books, original works, case reports and meta-analyzes, and the exclusion criterion was literature review. Concerning the publication time, articles were screened between 1960 and May 2015.

**Results:**

The dumping syndrome is complication arising from obesity surgeries, but also can be a result of vagus nerve damage. Diagnosis is done primarily through the use of questionnaires based on scores.

**Conclusion:**

The Sigstad score and Arts survey are valid means for assessing the dumping syndrome. Initial therapy consists in the adoption of dietary measures, short acting drugs administration.

## RESUMO

**Introdução:**

A síndrome de dumping é frequente após operações bariátricas. É, provavelmente, a mais comum das síndromes que sucedem gastrectomias parciais ou completas. Sua prevalência, em gastrectomias parciais pode chegar a até 50%, tornando-se assim complicação significante em alguns tipos de operações bariátricas.

**Objetivo:**

Realizar análise crítica sobre a síndrome de dumping em sua fisiopatologia, diagnóstico e tratamento.

**Métodos:**

Foi realizada revisão bibliográfica utilizando os descritores: 'síndrome de dumping', 'cirurgia bariátrica' e 'síndrome do esvaziamento rápido'. Os critérios de inclusão foram: livros, trabalhos originais, relatos de caso e metanálises; excluíram-se as revisões bibliográficas. Quanto ao tempo de publicação, foram selecionados artigos entre 1960 e maio de 2015.

**Resultados:**

A síndrome de dumping é complicação gastrointestinal oriunda de operações para obesidade, mas também pode ocasionar-se como consequência de danos no nervo vago. Seu diagnóstico é realizado primordialmente através da aplicação de questionários baseados em pontuações.

**Conclusão:**

O escore de Sigstad e questionário de Arts são meios válidos para avaliação da síndrome de dumping. A terapia inicial consiste na adoção de medidas dietéticas, ou fármacos administráveis de ação curta.

## INTRODUCTION

Obesity is caused by disturbances in energy metabolism and results in excessive storage of fat, leading to physiological and psychological problems^8^. Since 80's decade, it affects people of all ages and socioeconomic groups and its incidence has increased over the years^17^pharmacological, and surgical interventions, all of which can result in a reduction in obesity-related comorbidities and improvements in quality of life. However, subsequent weight regain often reduces the durability of these improvements. The objective of this article is to review evidence supporting the long-term effects of intentional weight loss on morbidity, mortality, quality of life, and health-care cost. Overall, considerable evidence suggests that intentional weight loss is associated with clinically relevant benefits for the majority of obesity-related comorbidities. However, the degree of weight loss that must be achieved and sustained to reap these benefits varies widely between comorbidities. In the United States, the percentage of obese and overweight adults is 22.3 and 54.9%, respectively. In 2006 the World Health Organization has recorded more than 400 million obese and 1.6 billion overweight people in the world and it is estimated that by 2015 these numbers will move to 700 million and 2.3 billion, respectively^3^.

Conventional treatments for the control of obesity, such as medicines, diets and regular physical activity, often do not have the success and the expected speed, bringing sense of failure^23^. On the other hand, bariatric surgery appears as permanent treatment, each day safer, less invasive, rapid recovery, and potential cure for several comorbidities^15^. Because of the importance of this procedure for the control of obesity, it is necessary to undertake studies focusing on adverse events experienced by patients after its completion^13^.

This review aims to conduct critical analysis about the dumping syndrome in its pathophysiology, diagnosis and treatment.

## METHODS

The literature was reviewed between January and May 2015. Data were collected through research from content that addressed the dumping syndrome comprised between 1960 and May 2015, available in books, dissertations, theses and published articles on Google Books, Scielo, PubMed, Lilacs and Bireme. The following descriptors were used: síndrome de dumping, doenças do sistema digestivo, cirurgia bariátrica, dumping syndrome, digestive system diseases, bariatric surgery. Inclusion criteria were: original articles, case reports and meta-analyzes; were excluded literature reviews.

## RESULTS 

Were analyzed 48 references, of which 26 were excluded by selective and analytical reading due to not being specifically about dumping syndrome, not fitting the inclusion criteria, containing irrelevant information to the research or being in the exclusion criteria, leaving 22 articles. Follow the highlights of the references that were selected for this study into the three areas focused as goals.

### Pathophysiology

The dumping syndrome may be present on bariatric surgical procedures; its prevalence may reach up to 50% in partial gastrectomy. It is probably the most common syndrome that follow gastrectomy. It was first described in 1913 by Hertz, correlating the symptoms with the accelerated gastric emptying; the term ''dumping'' was introduced by Mix in 1922 after recognizing the rapid gastric emptying in these conditions showed in contrasted radiographic examinations^12^.

It is characterized by a set of vasomotor and gastrointestinal symptoms associated with rapid gastric emptying or sudden nutrients exposure to the small intestine. It occurs after complete or partial gastrectomy, thereby becoming a significant complication arising from certain types of bariatric procedures involving gastrectomy; it also can happens as a consequence of damage to the vagus nerve^5^ ^,^ ^19^.

The sudden presence of gastric contents in the proximal small intestine has the physiological response to release of bradykinin, serotonin and enteroglucagon, together with the extracellular fluid, leading to early symptoms (need to lie down, palpitations, hypotension, tachycardia, fatigue, dizziness, sweating, headache, flushing, heat sensation of satiety, epigastric pain and fullness, diarrhea, nausea, vomiting, cramps, bloating, and borborygmus) in less than 30 min. Within 90 min to 3 h, the late symptoms (sweating, tremor, trouble concentrating, loss of consciousness and hunger) appears due to high insulin secretion causing hypoglycemia^6^.

Abell and Minocha^1^with body mass indexes exceeding 35 to 40, are often refractory to all therapies other than surgery. The increasing number of patients undergoing bariatric surgery will result in increasing numbers of patients with gastrointestinal complications. The types of complications vary with type of surgery, whether restrictive, malabsorptive, or both, depending on what anatomical and physiologic changes occur postoperatively. One complication of bariatric surgery (gallstones reported dumping symptoms arising in operations to treat obesity. It occurs more often after combined techniques and may have gastrointestinal and autonomic symptoms, early or late manifested. Early symptoms involve osmolar effect due to rapid emptying of stomach contents into the small intestine, gastrointestinal symptoms such as nausea, cramping and diarrhea. Late vasomotor symptoms are related to increased levels of insulin, followed by reactive hypoglycemia, although other hormones, such as glucagon, can be involved. 

Tack and Deloose^19^ reported that the dumping syndrome is characterized by a set of vasomotor and gastrointestinal symptoms associated with rapid gastric emptying or sudden exposure nutrients to the small intestine. It occurs after complete or partial gastrectomy, becoming so significant complication arising from bariatric surgery involving gastrectomy. Loss et al*.* ^12^ pointed frequency ranging from 1-75% and 25-30% in total and partial gastrectomy, respectively. With regard to Y-de-Roux gastric bypass the incidence of dumping symptoms can reach 75.9%. 

Amdrup^2^, in turn, showed that the syndrome may occur in patients who did not undergo gastric operations; however, the pylorus, somehow protects our body against these symptoms. In cases of post-gastrectomy dumping syndrome, prevalence can reach up to 50%. Ferguson *et al.* ^7^ concluded that the incidence was lower for resection techniques segment in which the pylorus was kept.

Zagury et al. ^22^ described the dumping syndrome as a beneficial side effect, since, it would help in weight loss in patients who undergo gastric operations, which tend to limit and restrict food intake. Schauer and Marema apud Loss^12^, associated the weight regained presented by patients followed for two to three years to the amelioration of the symptoms of dumping. Keshishian et al.^10^there has been a rise in the number of patients who have had less than desirable outcome after bariatric operations. We perform the duodenal switch (DS found an incidence of 28% of severe dumping studying 47 patients submitted to Y-de-Roux gastric bypass, and all had weight regained, contradicting the idea that dumping syndrome could help in weight loss.

Michaud et al.^14^ reported that surgical treatment of gastroesophageal reflux is the main dumping cause in childhood, although other rare causes exist or even meals intake with high osmolarity. Holschneider et al.^9^ reported cases where children with esophageal atresia present the dumping syndrome with absence of the main factors, such as operation for gastroesophageal reflux. Experimental studies in rats with esophageal atresia conducted by Tugay *et al.* ^20^ diagnose a defect in the contraction of the gastric fundus muscles, resulting in delayed gastric emptying.

### Diagnosis

The clinical diagnosis was only possible after 1970. Sigstad^18^ sought to establish criteria that could allow the differentiation of symptoms, since till then all symptoms presented by the gastrectomized patients were classified as dumpers. Through the correlation between the symptoms and the reduction of plasma volume, the author established association of symptoms with greater decrease in plasma volume, stablishing symptom score, making it possible to clinically diagnose dumpers and non-dumpers^12^.

Diagnostic questionnaires were created based on symptoms, such as Sigstad Score Scale and the Arts Dumping Questionnaire, to identify clinically significant symptoms^19^.

Sigstad scoring system is based on the occurrence of various symptoms suggestive of the syndrome. Scores greater than or equal to seven, after glucose intake, are considered diagnostic^21^.

**FIGURE 1:**
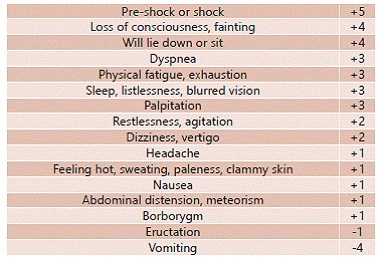
Sigstad score: value applied to the signs and symptoms of dumping syndrome^18^

In 1970, Sigstad^18^ also showed a way to establish criteria that would allow the clinical diagnosis of the syndrome, since until then all symptoms after gastrectomy were taken as such. By correlating the symptoms reported by patients and reduction of its plasma volume, has been drawn associated symptoms, so that the higher the drop presented by the plasma volume, higher the index determined by the score symptoms. Based on this score (Figure 1) turned possible to distinguish patients clinically dumpers or non-dumpers by obtaining values greater than or equal to seven and less than seven, respectively.

**FIGURE 2:**
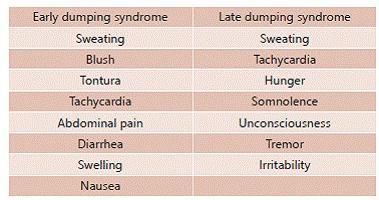
Arts score: signs and symptoms to be evaluated according to their intensity^21^

The Arts score (Figure 2) is based on the assessment of the severity of symptoms after ingestion of glucose for the first time for diagnosis of early dumping, and one to two hours to late dumping. The score was developed using the Likert scale of four points for standard symptoms of dumping crisis, enabling the classification of its intensity on a scale of 0-3, where 0 represents the absence of certain symptoms, 1 mild, 2 moderate and 3 severe intensity^21^.

Loss et al.^12^, protocol analyzed 34 patients, 30 men and four women ages ranging from 21-62 years, and initial weights of 92-180 kg and 52-143 kg, before and after performing some kind of operation to obesity, respectively. 

Before applying the score of the syndrome, when questioned, 44% denied having it, while among the non-dumpers 16% believed to have. By applying, the Sigstad score was observed that the frequency of the dumping syndrome based on subjective criteria was 44%, whereas, by the score, the value reached 76%. 

Diagnosis confirmation is obtained by oral glucose tolerance test, which administer 50 g of glucose in water, evaluating blood glucose, hematocrit and the pulse for 3 h at 30 min intervals. The diagnosis is positive if there is initial hyperglycemia and final hypoglycemia (<60 mg/dl or 3.33 mmol/l), initial increase in hematocrit in more than 3% or increase in initial pulse rate greater than 10 bpm^19^.

 In addition to the history, application of the Sigstad score and the glucose tolerance test, are used in dumping syndrome diagnosis fasting test, HbA1C levels, scintigraphy with monitored food, quantification of breath and acid breath test. For differential diagnosis can be done administration of exogenous insulin, sulfonylureas measurement, and proinsulin C-peptide, pancreatic proof image and selective arterial stimulation with calcium for determination of insulin^16^.

In the study by Loss *et al.* ^12^, 80% of dumper patients claimed improvement of symptoms after about 30 min of its beginning; of these, 20% said they stimulate vomiting, 63% had adopted the supine position and 70% said they adopt changes in diet to avoid or mitigate the symptoms. 

### Treatment

The dumping syndrome treatment is based on delaying gastric emptying^4^000 large obeses underwent bariatric surgery in 2010. The surgery technique and consequent altered gastrointestinal function done will particularly imply in variations on those deficiencies and health complications. A systematic review of several database was done from 1978 until 2010, using as keywords protein and micronutrients deficiencies, related to bariatric surgery. The better understand of these studies can provide an important improvement on this obese therapy, assuring a successful and health weight loss maintenance for long term. Therefore, this review provides a significant contribution about this topic, pointing several ways on the nutritional intervention and management of those patients. For patients with mild to moderate attacks, change in diet is beneficial. Pectin, glucomannan, alpha-glucosidase inhibitor and acarbose, may also be useful for prolonged carbohydrate absorption, reducing postprandial glucose and insulin production^11^.

As the initial therapy consists in the adoption of dietary measures, patients are advised to carry smaller meals more often (up to six per day), avoid fluid intake during meals or within the first 2 h after. Moreover, they are oriented to avoid sugars of fast absorption and lactose. Other options may be considered such as the use of viscous food additives - pectin, guar gum and glucomannan - along with meals, to delay gastric emptying; however, they are referred to be unpleasant and less effective in the case of partial gastrectomy. Acarbose - intestinal alpha-glucosidase inhibitor - can also be used to delay the digestion of carbohydrates, but its use is limited by lack of efficacy for early symptoms, and the occurrence of side effects of carbohydrates and poor digestion, such as flatulence and diarrhea. In the absence of initial efficacy, can be used somatostatin analogues; these agents are considered the most effective treatments for both symptoms, early and late. They can be administered by subcutaneous (three times daily) for early syndrome or intramuscularly every two or four weeks for late symptoms, due to release inhibition of numerous gastrointestinal peptides^19^.

As more patients undergo bariatric surgery as alternative option for morbid obesity, greater is the increase for revision of these procedures^10^there has been a rise in the number of patients who have had less than desirable outcome after bariatric operations. We perform the duodenal switch (DS).

Initial therapy is made by subcutaneous administration of short-acting agents - for example, 50 to 100 μg octreotide -, and in case of efficacy and tolerance, a dose of slow-release 20 mg can be administered intramuscularly. In more severe cases, can be considered surgical methods such as the reconstruction of a gastric reservoir, add restrictive intervention, undo the operation or, if possible, insert a short antiperistaltic loop^11^.

Reconstructive operations are treatment alternatives, but only reserved for severely affected patients. However, their results often are irrelevant and of limited effectiveness^11^. The review of any bariatric surgery for dumping syndrome, involves detailed study of the existing anatomy including review of previous medical records, when available, as well as thorough clinical examination followed by upper gastrointestinal examinations^10^there has been a rise in the number of patients who have had less than desirable outcome after bariatric operations. We perform the duodenal switch (DS).

The Brazilian Society of Bariatric and Metabolic Surgery claims that the demand for surgical procedures to combat obesity is growing about 20% per year in Brazil; so, it is important to direct actions aiming to aware the population about the hardships to which they are exposed, where dumping syndrome is included.

## CONCLUSION

Although established in previous studies the correlation between change in plasma volume and blood volume, the predisposing factors for the development of the dumping syndrome after partial or total gastrectomy are not clear. The impact of rapid gastric emptying has been highlighted, but it is important to clarify that not all patients with accelerated gastric emptying suffers from dumping. This phenomenon is due to functional sequel procedures in the upper gastrointestinal tract, especially when alter gastric anatomy, and is still not well understood. The accelerated gastric emptying of hypertonic substances results in osmotic change in fluids, promoting the symptoms.

Questionnaires (score of Sigstad and Arts questionnaire) based on intensity rating scores and occurrence of symptoms are the main diagnostic methods.

Early treatment is easily performed, since it is based on dietary change. The cases in which crises are moderate or severe drug therapy or even surgery may be required. Surgical treatment is reserved only to treat severely affected patients, with intense and disabling symptoms.
